# Statistical modeling of volume of alcohol exposure for epidemiological studies of population health: the US example

**DOI:** 10.1186/1478-7954-8-3

**Published:** 2010-03-04

**Authors:** Jürgen Rehm, Tara Kehoe, Gerrit Gmel, Fred Stinson, Bridget Grant, Gerhard Gmel

**Affiliations:** 1 Centre for Addiction and Mental Health (CAMH), 33 Russell Street, Toronto, Ontario, M5S 2S1, Canada; 2 Dalla Lana School of Public Health, University of Toronto, 6th Floor, Health Sciences Building, 155 College Street, Toronto, Ontario, M5T 3M7, Canada; 3 Institute for Clinical Psychology and Psychotherapy, Technische Universität Dresden, Chemnitzer Str. 46 D-01187 Dresden, Germany; 4 Department of Statistics, University of Toronto, 100 St. George St, Toronto, Ontario, M5S 3G3, Canada; 5 National Institute on Alcohol Abuse and Alcoholism/NIH, Laboratory of Epidemiology and Biometry, Division of Intramural Clinical and Biological Research, 5635 Fishers Lane, Rockville MD 20852, USA; 6 Swiss Institute for the Prevention of Alcohol and Drug Problems PO Box 870, 1001 Lausanne, Switzerland; 7 Alcohol Treatment Center, Lausanne University Hospital, Mont-Paisible 16, 1011 Lausanne, Switzerland; 8 University of the West of England, Frenchay Campus Coldharbour Lane, Bristol BS16 1QY, UK

## Abstract

**Background:**

Alcohol consumption is a major risk factor in the global burden of disease, with overall volume of exposure as the principal underlying dimension. Two main sources of data on volume of alcohol exposure are available: surveys and per capita consumption derived from routine statistics such as taxation. As both sources have significant problems, this paper presents an approach that triangulates information from both sources into disaggregated estimates in line with the overall level of per capita consumption.

**Methods:**

A modeling approach was applied to the US using data from a large and representative survey, the National Epidemiologic Survey on Alcohol and Related Conditions. Different distributions (log-normal, gamma, Weibull) were used to model consumption among drinkers in subgroups defined by sex, age, and ethnicity. The gamma distribution was used to shift the fitted distributions in line with the overall volume as derived from per capita estimates. Implications for alcohol-attributable fractions were presented, using liver cirrhosis as an example.

**Results:**

The triangulation of survey data with aggregated per capita consumption data proved feasible and allowed for modeling of alcohol exposure disaggregated by sex, age, and ethnicity. These models can be used in combination with risk relations for burden of disease calculations. Sensitivity analyses showed that the gamma distribution chosen yielded very similar results in terms of fit and alcohol-attributable mortality as the other tested distributions.

**Conclusions:**

Modeling alcohol consumption via the gamma distribution was feasible. To further refine this approach, research should focus on the main assumptions underlying the approach to explore differences between volume estimates derived from surveys and per capita consumption figures.

## Introduction

The volume of alcohol consumed has been shown to be causally related to more than 230 International Classification of Disease, version 10, disease codes [1-3]. Most of these relationships follow the component cause model [4], in which only a fraction (the so-called attributable fraction [5]), of the incidence of a disease would disappear if the causal component, in this case alcohol use [6], could be eliminated. Liver cirrhosis may be used as an example: alcohol use has been shown to have a causal impact on liver cirrhosis [7-9]; however, there are also cases of liver cirrhosis where alcohol use has not been involved, such as those stemming from HCV infection in nondrinkers.

The proportion of most diseases caused by alcohol in the component cause model in a population is determined by:

• The distribution of the volume of exposure

• The relative risk associated with each level of exposure, i.e., dose-response relationship [10]

For most disease categories, the dose-response relationship is nonlinear and varies by sex as well as age in some cases. Thus, to calculate the alcohol-attributable fractions (AAFs) for estimating a population's burden of disease attributable to alcohol, we need to characterize the volume of alcohol exposure by sex and age.

There is one main problem with the characterization of the volume of alcohol exposure in populations: the best indicator, adult per capita consumption [11], is not available by sex and age. It is derived mainly from production, sales, export, and import figures, which are almost never disaggregated [12]. As an alternative, alcohol exposure can be measured from surveys, yet this has the disadvantage that adult per capita figures, the best indicator for consumption, are often severely underestimated [11]. To give but one recent example: the national Canadian Addiction Survey [13] had a coverage rate of between 30% and 40% of the adult per capita consumption. Thus, estimating overall volume of consumption in Canada based on answers from a representative survey results in a figure that is 60% to 70% lower than the figure derived from aggregate statistics mainly based on sales and taxation. The exact magnitude of underestimation depends on the alcohol measure used in the survey [14].

This underestimation of population exposure leads to two problems: first, the absolute level of exposure is incorrectly estimated, usually underestimated. And second, inter- and intrapopulation distributions based on different surveys are not adequately comparable as the degree of incorrect estimation will vary. Recent large, nationally representative surveys have shown coverage rates between 25% and more than 100% [15]. Studies have thus had to explore additional means for achieving comparability. In the case of the Comparative Risk Assessment (CRA) for alcohol within the Global Burden of Disease (GBD) 2000 study, this was achieved by triangulating survey and per capita information based on sales or production [12,15]. In short, the distribution of volume by sex and age was taken from surveys, while the overall exposure was taken from adult per capita figures [15].

There is, however, more than one way to triangulate such data. The CRA 2000, for instance, used a categorical approach based on the standard categories of volume of drinking from English and colleagues [16]. For shifting the distribution of the survey to correspond to the per capita consumption, however, an additional assumption had to be made to obtain a unique solution, and the assumption chosen at the time was that the highest drinking category was a constant fraction of the percentage of the next highest category [15]. However, no empirical evidence supports this specific assumption. Therefore, a more evidence-based approach was sought for the ongoing CRA of the GBD 2005 study. Different distributions (gamma, log-normal, Weibull) were fitted to empirical data of surveys from 66 countries with the aim of identifying associations between distribution parameters that would enable shifting survey distributions to fit the volume of drinking indicated by adult per capita consumption data [17]. The two-parameter gamma distribution proved to be best suited for triangulating survey and per capita data by shifting distributions upward because:

1) As an inherent characteristic of this distribution, the means of fitted distributions are equal to those of the empirical distributions. Thus, there is no error involved in estimating means from the fitted distribution and no need for erroneous and complicated back-transformations from fitted distributions to original scales (as in the case of the log-normal distribution).

2) There was a very high correlation between means and standard deviations of the sex- and age-specific, fitted two-parameter gamma distributions (r = 0.923; N = 851; p < 0.01). We then conducted a linear regression with the standard deviation as the dependent variable, and mean and sex as the independent variables, and could predict the standard deviation with precision (r = 0.971; N = 851; p < 0.01; for the derived prediction equation, see below). Thus, the shifted distributions could easily and reliably be derived from the mean (= per capita consumption) and the standard deviation as estimated by regression methods [17].

This article discusses the modeling approach described above in its application to US data, with three main objectives:

1. To model the volume of alcohol exposure in the US with three different distributions: log-normal, gamma, and Weibull distribution for different strata by sex, age and ethnicity. The volume of alcohol use was obtained from a large representative survey, the National Epidemiologic Survey on Alcohol and Related Conditions (NESARC).

2. To shift the alcohol use distribution to the level of adult per capita consumption.

3. To show the impact of shifting the alcohol use distribution on AAFs, using liver cirrhosis as an example.

## Methods

### Description of underlying survey (NESARC)

This analysis is based on data from the 2001-2002 NESARC, which was designed and sponsored by the National Institute on Alcohol Abuse and Alcoholism. The fieldwork for the NESARC was conducted by the US Census Bureau, with data collected in face-to-face, computer-assisted, in-home interviews. The NESARC sample represents the civilian, noninstitutionalized adult population of the United States, including the District of Columbia, Alaska, and Hawaii, and includes people living in households and military personnel living on and off base [18]. The NESARC oversampled African Americans, Hispanics, and adults aged 18 to 24. One sample adult (age 18 or older) was selected for an interview in each household. The overall response rate was 81% (N = 43,093).

The volume of ethanol consumption reflected consumption in the 12 months preceding the interview. The volume of ethanol intake was based on data summed over a separate series of questions for coolers, beer, wine, and distilled spirits. For each beverage, volume was estimated on the basis of: overall frequency of drinking; typical and largest quantities consumed; frequency of consuming the largest quantity; frequency of consuming five-plus drinks; typical drink size; and ethanol content by volume of the brand usually consumed. The test-retest reliabilities for the various measures of alcohol consumption from the 2001 to 2002 NESARC were good to excellent, with intraclass correlation coefficients ranging from 0.68 to 0.84 [19].

### Methods for fitting the distributions

To find an appropriate model for alcohol consumption, we examined three distributions that were unimodal, had a density with only one maximum, and could be used to fit right-skewed empirical data: log-normal, gamma, and Weibull. The log-normal, gamma, and Weibull densities are similar in shape and mainly differ at the tails (i.e., at high levels of consumption). Alcohol consumption has been more commonly modeled using the log-normal distribution, mostly for historical reasons related to the so-called single distribution theory [20,21]. But the log-normal distribution also has been favored because it is easy to use as a transformation and results in sufficiently accurate values that permit fitting and testing hypotheses [22]. Although doubts regarding the single parametric log-normal distribution and its justification as the best approximation for the distribution of consumption have been previously raised, [23,24] it has appeared to provide good approximations for most applications [25,26]. Later developments on modeling alcohol exposure have favored more complex distributions such as gamma [27], or revealed that mixing distributions is needed to fit separate distributions for frequency of drinking and quantity of drinking [28].

The log-normal distribution, with parameters *μ *and *σ*, describes a random variable *X *where log *x *is normally distributed with mean *μ *and standard deviation *σ*. The log-normal distribution function, with parameters *μ *and *σ*, is given by:

Although alcohol consumption is frequently modeled using the log-normal distribution, empirical distributions often deviate considerably from the log-normal model [27-29].

The gamma distribution has two parameters, a scale parameter *θ *and a shape parameter *k*. The gamma distribution is more adaptable than the log-normal distribution because it has the effect of stretching or compressing its range by changing the scale parameter *θ*. The gamma distribution has the following probability density function:

The Weibull distribution is one of the most widely used distributions in applied statistics, especially life data analysis, because of its versatility in fitting a variety of distributions. The probability density function of a random variable *X *having a two-parameter Weibull distribution with shape parameter *γ *and scale parameter *θ *is given by:

The shape parameter *γ *in the Weibull distribution gives this distribution its flexibility.

The maximum likelihood method of estimation was used to fit all three models -- log-normal, gamma, and Weibull -- to the data using the R language [30]. All missing values on volume of drinking -- 298 in total (men 185; women 113) -- were excluded from the fitted models. All numerical integration used the trapezoidal rule with many subintervals to obtain more accurate estimates. The trapezoidal rule uses trapezoids instead of rectangles for approximating the definite interval over closed bounded intervals. The Newton-Raphson algorithm, having a quadratic rate of convergence, was used to optimize the likelihood equations solving for the unknown parameters with maximum likelihood estimates.

To compare different fitted distributions, we used chi-square tests by comparing expected frequencies (derived from the fitted distributions) and observed frequencies from the empirical distributions, using a bandwidth of 10 grams of ethanol for the frequencies. The use of chi-square tests to compare two distributions is a standard method [31].

### Method for shifting the distribution

As indicated above, the gamma distribution has two parameters *k *and *θ*, which can be expressed in terms of means and standard deviations using the intrinsic properties of the gamma function, namely:

hence

The gamma distribution also has the welcome property of its mean being the same as that of the empirical distribution. To shift a gamma distribution, the mean and standard deviation of the shifted distribution must be known. However, per capita consumption only indicates the mean of the (up-)shifted distribution. To derive the standard deviation of this distribution, we had to find a way to predict the standard deviation based on the mean. This was achieved via regression from the large global dataset.

To shift the gamma functions to fit the adult per capita level, two crucial assumptions had to be made:

1. The proportion of abstainer categories as derived from the survey reflected the true proportion of current abstainers (lifetime abstainers plus ex-drinkers) in the population.

2. The overall coverage rate for the survey (i.e., the total volume of alcohol exposure derived from the survey divided by the adult per capita consumption from sales or other statistics) applied to all subpopulations as defined by age and sex.

We found a coverage rate of 0.529% between the NESARC survey and adult per capita alcohol consumption for the US, estimated at 8.75 liters per capita for 2001-2002 when NESARC took place based on the Global Information System for Alcohol and Health http://apps.who.int/globalatlas/default.asp. Using this value, the shifted means of the drinkers for different subpopulations as described by age, sex, and ethnicity were derived as follows with the same constant for all subpopulations:

As described above, the shifted standard deviation was derived empirically via regression analyses [17]:

where sex was coded 0 for men and 1 for women. In the regression, N = 851 sex and age subpopulations from 66 countries, individually modeled to derive the above equation [17]. The multiple linear regression based on all these surveys explained 94% of variation of the dependent variable.

### Method for deriving AAFs

Knowing the percentages of abstainers and former drinkers, as well as the risk relation, the AAFs based on continuous distributions were obtained using the following formula:

while the categorical value of the AAFs were obtained using:

where P_abs _represents the proportion of lifetime abstainers, P_form _the proportion of former drinkers, and P(x) the probability distribution function of drinkers. RR_form _represents the relative risk for former drinkers, and RR(x) the relative risk function for a given alcohol consumption in grams per day. The subscript i denotes the groups as characterized by different categories for volume of drinking. We conducted a sensitivity analysis with consumption capped at 150 grams of pure alcohol per day.

Confidence intervals were based on simulations using the bootstrapping method [32]. For each AAF, 10,000 simulations were run.

## Results

Additional File 1 gives an overview of the volume of drinking for different groups as defined by sex, age, and ethnicity. Overall, as expected, the younger the age group among adults, the higher the volume. Men consumed more than women, and Native Americans consumed on average more than other ethnicities.

**Figure 1:**
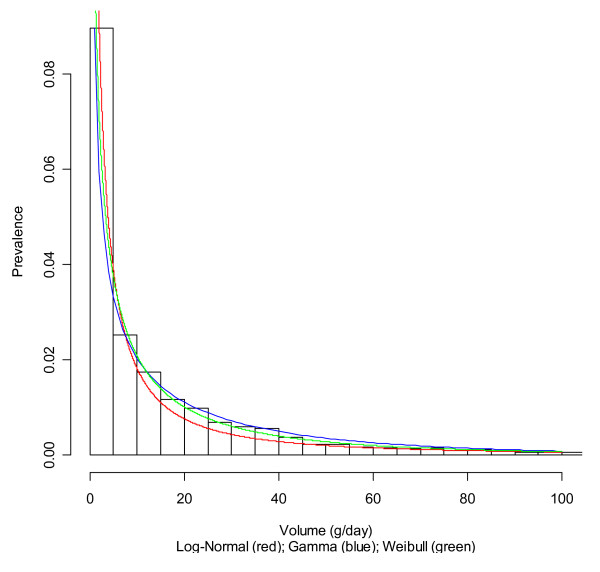
**Histogram of alcohol exposure and fitted distributions for non-Hispanic white men**.

**Figure 2:**
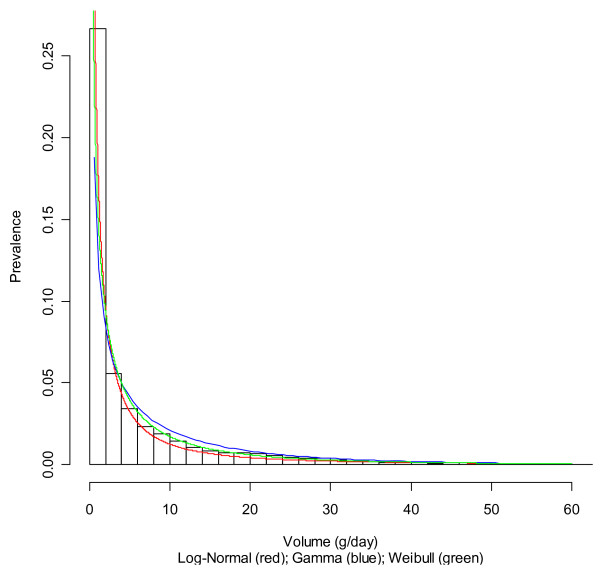
**Histogram of alcohol exposure and fitted distributions for non-Hispanic white women**.

Table 1 and Figures 1 and 2 show the fitting of different distributions to the data for non-Hispanic whites as an example. All three distributions fit the data reasonably well, with some deviations mainly in the tails of the distributions. For consumption levels up to 100 grams, the Weibull and gamma distributions were very similar and fit well, but the log-normal distribution underestimated drinking at lower levels of drinking.

**Table 1:** Example for fitting exposure distributions for white non-Hispanic Americans

Goodness of fit							Chi Square	
Volume of drinking in g/day	Count	Empirical distribution %	log-normal fit %	gamma fit %	Weibull fit %	log-normal	gamma	Weibull
Men								
0 - 10	4524	57.3	64.6	52.2	57.3	73.0	53.3	0.5
10 - 20.	1163	14.7	11.7	15.2	14.6	50.4	0.2	0.5
20 - 30	666	8.4	5.8	9.2	7.9	67.5	2.6	3.3
30 - 40	459	5.8	3.5	6.1	5.0	70.9	0.5	10.3
40 - 50	238	3.0	2.4	4.3	3.4	9.4	38.2	3.2
50 - 60	156	2.0	1.8	3.1	2.4	1.7	47.0	7.3
60 - 70	119	1.5	1.4	2.3	1.8	1.2	29.8	3.8
70 - 80	95	1.2	1.1	1.7	1.4	1.1	15.8	1.3
80 - 90	85	1.1	0.9	1.3	1.1	3.1	3.1	0.1
90 - 100	48	0.6	0.7	1.0	0.8	1.7	17.8	5.7
100 +	336	4.3	6.5	3.5	4.0	96.9	13.3	1.9
**TOTAL**	**7889**	**100.0**	**100.0**	**100.0**	**100.0**	**377.0**	**221.6**	**37.7**
Women								
0 - 10	6908	79.7	82.8	74.5	79.6	10.7	60.1	3.2
10 - 20.	848	9.8	7.3	13.8	10.5	53.0	116.2	2.4
20 - 30	443	5.1	3.1	6.0	4.3	69.5	8.3	14.4
30 - 40	169	1.9	1.7	2.9	2.2	2.5	35.0	1.2
40 - 50	92	1.1	1.1	1.5	1.2	0.1	13.2	1.4
50 - 60	65	0.7	0.8	0.8	0.7	0.0	0.1	0.1
60 - 70	37	0.4	0.5	0.4	0.5	3.1	0.0	0.2
70 - 80	16	0.2	0.4	0.2	0.3	25.5	1.2	6.6
80 - 90	19	0.2	0.3	0.1	0.2	4.6	3.1	0.1
90 - 100	17	0.2	0.3	0.1	0.1	2.0	6.6	1.2
100 +	56	0.6	1.8	0.1	0.4	191.6	40.3	7.2
**TOTAL**	**8670**	**100.0**	**100.0**	**100.0**	**100.0**	**362.4**	**284.0**	**37.8**

Table 2 gives an overview of all the chi-square deviations for the different subgroups as defined by sex, age, and ethnicity. Clearly, the Weibull distribution fits best, but there are no options for easily shifting this distribution [17]. However, for descriptive purposes only, the Weibull distribution seems to best fit the various distributions. For shifting distributions, the gamma distribution was chosen mainly for feasibility. However, it offered a relatively good fit for descriptive purposes as well. Overall, for several subgroups, gamma had a better fit than log-normal, whereas for others, log-normal showed the better fit (Table 2).

**Table 2:** Fit indices for different distributions

Sex	Ethnicity	Age category	Chi-square total			Chi-square up to 100 g pure alcohol per day		
			log-normal	gamma	Weibull	log-normal	gamma	Weibull
Men	White	18 - 34	83.2	89.7	22.9	68.0	89.3	22.6
Men	White	35 - 54	108.1	136.4	24.0	93.0	123.5	18.9
Men	White	55+	174.5	23.6	25.0	136.0	21.5	23.6
Men	Black	18 - 34	19.7	47.8	13.3	17.7	47.8	13.3
Men	Black	35 - 54	42.4	56.6	22.7	38.6	56.4	22.7
Men	Black	55+	14.2	13.2	0.7	12.5	13.0	0.6
Men	Native	18 - 34	8.6	27.6	15.0	8.6	25.8	14.9
Men	Native	35 - 54	9.6	21.5	11.9	8.7	21.1	11.2
Men	Native	55+	3.8	5.7	3.3	3.0	5.0	2.5
Men	Asian/Pac. Islander	18 - 34	4.3	18.9	6.8	4.2	16.8	5.6
Men	Asian/Pac. Islander	35 - 54	7.1	28.0	12.6	6.6	26.4	11.5
Men	Asian/Pac. Islander	55+	5.0	11.2	6.2	4.5	10.7	5.7
Men	Hispanic	18 - 34	21.2	94.4	20.4	21.2	88.7	16.9
Men	Hispanic	35 - 54	42.9	63.9	14.2	27.3	62.1	13.8
Men	Hispanic	55+	8.9	21.3	5.5	8.5	18.7	4.7
Women	White	18 - 34	42.4	243.0	53.0	29.5	221.7	43.6
Women	White	35 - 54	206.0	81.0	27.1	122.6	68.5	25.7
Women	White	55+	220.9	44.6	28.2	102.9	39.8	27.5
Women	Black	18 - 34	9.9	116.8	15.9	9.8	111.1	13.1
Women	Black	35 - 54	33.5	58.5	8.1	26.7	53.8	7.2
Women	Black	55+	9.3	24.0	4.7	9.2	22.4	4.2
Women	Native	18 - 34	4.2	18.1	6.2	3.9	18.1	6.0
Women	Native	35 - 54	7.4	26.5	11.7	6.8	25.7	10.9
Women	Native	55+	3.4	5.6	3.2	2.9	5.1	2.7
Women	Asian/Pac. Islander	18 - 34	3.2	49.5	14.0	3.0	48.8	13.1
Women	Asian/Pac. Islander	35 - 54	6.0	4.3	4.8	5.5	3.8	4.3
Women	Asian/Pac. Islander	55+	5.4	4.8	5.1	4.9	4.3	4.6
Women	Hispanic	18 - 34	9.1	140.3	17.6	8.4	133.8	14.4
Women	Hispanic	35 - 54	13.7	34.4	6.6	12.9	29.5	2.8
Women	Hispanic	55+	6.7	10.2	3.5	6.4	8.3	2.0

**Figure 3:**
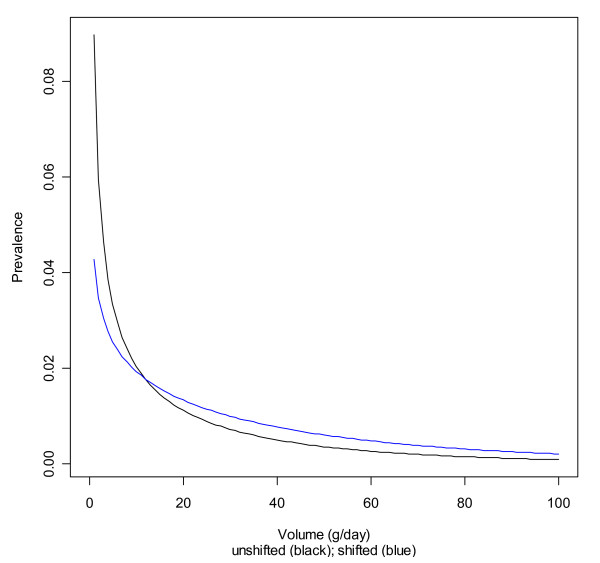
**Original and shifted gamma distributions for non-Hispanic white men**.

**Figure 4:**
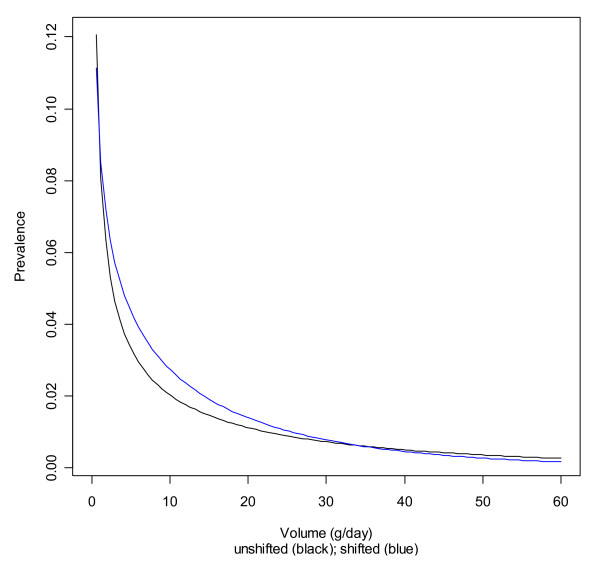
**Original and shifted gamma distributions for non-Hispanic white women**.

Table 3 lists the parameters of the original and the shifted gamma distributions. Again, the unshifted and shifted distributions for non-Hispanic whites are given for illustration in Figures 3 and 4.

**Table 3:** Parameter estimates for original and shifted gamma distributions

Sex	Ethnicity	Age	Original NESARC distribution		Upshifted distribution			
			k	Theta	Mean	SD	K	Theta
Men	White	All Ages	0.436	48.132	40.268	47.275	0.726	55.501
Men	White	18 - 34	0.450	58.550	50.586	59.388	0.726	69.721
Men	White	35 - 54	0.441	46.951	38.993	45.778	0.726	53.743
Men	White	55+	0.430	37.486	29.713	34.883	0.726	40.953
Men	Black	All Ages	0.387	65.821	49.570	58.195	0.726	68.321
Men	Black	18 - 34	0.380	65.850	47.957	56.301	0.726	66.098
Men	Black	35 - 54	0.406	65.155	52.860	62.058	0.726	72.856
Men	Black	55+	0.362	66.530	44.642	52.410	0.726	61.529
Men	Native	All Ages	0.354	88.399	56.528	66.363	0.726	77.911
Men	Native	18 - 34	0.354	111.130	70.687	82.987	0.726	97.427
Men	Native	35 - 54	0.376	87.400	58.111	68.223	0.726	80.094
Men	Native Asian/Pac.	55+	0.345	46.584	27.412	32.181	0.726	37.781
Men	Islander Asian/Pac.	All Ages	0.417	28.224	22.544	26.467	0.726	31.072
Men	Islander Asian/Pac.	18 - 34	0.460	27.784	26.022	30.549	0.726	35.865
Men	Islander Asian/Pac.	35 - 54	0.412	30.006	22.784	26.749	0.726	31.403
Men	Islander	55+	0.345	17.665	11.801	13.854	0.726	16.265
Men	Hispanic	All Ages	0.426	40.756	40.726	47.812	0.726	56.131
Men	Hispanic	18 - 34	0.422	43.417	36.148	42.438	0.726	49.823
Men	Hispanic	35 - 54	0.433	40.633	52.215	61.300	0.726	71.967
Men	Hispanic	55+	0.426	31.678	23.517	27.609	0.726	32.413
Women	White	All Ages	0.391	19.778	14.785	18.361	0.648	22.801
Women	White	18 - 34	0.399	23.009	17.353	21.376	0.659	26.330
Women	White	35 - 54	0.410	18.282	14.224	17.702	0.646	22.030
Women	White	55+	0.366	17.852	12.495	15.672	0.636	19.657
Women	Black	All Ages	0.316	27.729	20.069	24.565	0.668	30.066
Women	Black	18 - 34	0.311	30.996	19.844	24.299	0.667	29.756
Women	Black	35 - 54	0.326	27.461	22.642	27.585	0.674	33.607
Women	Black	55+	0.310	19.494	12.313	15.459	0.634	19.408
Women	Native	All Ages	0.295	54.514	33.405	40.221	0.690	48.427
Women	Native	18 - 34	0.297	87.190	49.135	58.688	0.701	70.098
Women	Native	35 - 54	0.304	42.472	27.347	33.109	0.682	40.084
Women	Native Asian/Pac.	55+	0.336	20.350	19.866	24.326	0.667	29.787
Women	Islander Asian/Pac.	All Ages	0.318	24.855	14.464	17.984	0.647	22.360
Women	Islander Asian/Pac.	18 - 34	0.311	42.172	24.428	29.682	0.677	36.065
Women	Islander Asian/Pac.	35 - 54	0.441	6.702	5.460	7.413	0.542	10.065
Women	Islander	55+	0.360	5.835	2.430	3.856	0.397	6.118
Women	Hispanic	All Ages	0.341	17.855	18.482	22.701	0.663	27.883
Women	Hispanic	18 - 34	0.325	23.545	15.423	19.109	0.651	23.677
Women	Hispanic	35 - 54	0.377	12.815	24.680	29.977	0.678	36.411
Women	Hispanic	55+	0.330	13.513	10.682	13.544	0.622	17.173

Finally, the implications of the distributional shift for the AAFs of liver cirrhosis are displayed in Table 4. Clearly, the shift in distribution results in changes in AAFs that are public health-relevant. On the other hand, Table 4 also shows that the different distributions did not have a marked influence on AAFs, further justifying the choice of the gamma distribution.

**Table 4:** AAFs (in %) for liver cirrhosis for subpopulations as defined by sex and ethnicity based on different distributions

Sex	Ethnicity	Continuous AAF from NESARC using log normal	Continuous AAF from NESARC using Weibull	Continuous AAF from NESARC using gamma	Continuous AAF from shifted distribution*	Categorical AAF from NESARC**	Categorical AAF from shifted distribution**
Men	White	51.2	52.6	55.1	72.3	55.1	71.9
Men	Black	49.1	52.4	57.0	73.2	54.6	72.2
Men	Native	51.1	55.9	62.2	76.1	58.7	74.9
Men	Asian/Pac. Islander	34.7	31.4	30.9	49.7	37.5	52.3
Men	Hispanic	45.0	44.9	47.3	71.3	48.3	70.9
Women	White	66.2	65.2	65.7	73.5	73.6	77.2
Women	Black	65.1	65.3	66.9	75.1	72.0	77.2
Women	Native	70.3	71.6	74.6	82.3	76.8	83.1
Women	Asian/Pac. Islander	53.6	53.7	56.4	63.4	63.1	67.5
Women	Hispanic	56.8	56.2	57.8	71.7	67.4	74.6

* The gamma distributions were shifted upward to the level of the per capita data.

** Based on four drinking categories: > 0 - 30 g/day; > 30 - 60 g/day; > 60 - 90 g/day; > 90 g/day.

Furthermore, the differences between the continuous and the categorical approach were not pronounced. The results for the confidence intervals are listed in Table 5.

**Table 5:** AAFs (in %) for liver cirrhosis and corresponding 95% confidence intervals for subpopulations as defined by sex and ethnicity based on different distributions

Sex	Ethnicity	Continuous AAF from shifted distribution*	Standard Error	95% Confidence interval	
				Lower Bound	Upper Bound
Men	White	72.3	0.45	71.4	73.2
Men	Black	73.2	0.49	72.2	74.2
Men	Native	76.1	0.83	74.5	77.7
Men	Asian/Pac. Islander	49.7	0.98	47.8	51.6
Men	Hispanic	71.3	0.47	70.4	72.2
Women	White	73.5	0.24	73.0	74.0
Women	Black	75.1	0.26	74.6	75.6
Women	Native	82.3	0.45	81.4	83.2
Women	Asian/Pac. Islander	63.4	0.81	61.8	65.0
Women	Hispanic	71.7	0.34	71.0	72.4

* The gamma distributions were shifted upward to the level of the per capita data.

## Discussion

A procedure for triangulating survey and per capita data for deriving population exposure based on the gamma distribution for drinkers was presented and explored. This procedure proved feasible for modeling US drinking and generating AAFs based on the per capita consumption. It also allowed for the quantitative comparability of data on alcohol exposure from surveys with different coverage rates and a necessary correction for varying coverage rates.

One crucial assumption made in the triangulation process was a constant factor of underreporting for all subpopulations as defined by sex, age, and ethnicity (see formula for the shifted mean  above). There is no conclusive literature on differential underreporting by different subpopulations; even the literature on underreporting by volume of drinking is not conclusive [11]. It may be hypothesized that the more irregular the occasions of heavy drinking, the more underreporting there is as such occasions are difficult to report with our standard instruments. However, research is necessary to test this hypothesis before differential upshifting factors can be used for subpopulations. The methodological framework presented here certainly allows for such modifications.

One argument against upshifting the distribution of alcohol consumed to the level of per capita data is that the risk relations usually are also derived from self-reports on alcohol exposure, i.e., that the subject's responses in epidemiological studies also underestimate real drinking. There are, however, several counterarguments. First, alcohol exposure measurement in medical epidemiological studies and in general appears to yield valid individual consumption levels [33,34]. Second, as typically found in medical epidemiological studies [33], there are higher intercorrelations with external standards when alcohol is embedded into a series of other food items. Third, it has been shown that embedded alcohol items yield higher levels of consumption compared to questionnaires specifically targeting alcohol use [35]. Thus, there are indications that questions on alcohol in medical epidemiological studies yield more consistent and higher alcohol exposure compared to those in typical national alcohol surveys. However, the degree of difference between these approaches is not clear.

There are three principal ways to explain the undercoverage of per capita consumption in surveys:

• Measurement error in surveys due to sampling;

• Measurement error in surveys due to respondents' behavior, such as underreporting, problems in averaging, forgetting, or dropping out of the survey [11];

• Measurement error in per capita consumption.

It is beyond the scope of this article to give a full review on the types of measurement error in alcohol surveys. The literature is diverse and highly speculative, and there are few systematic studies on reasons for undercoverage. In high-income countries such as the US, there are groups left out of sampling frames who are very high alcohol consumers, such as the homeless or those living in institutions. Consumption is skewed, with a small segment of the population consuming a high proportion of alcohol. In the NESARC sample, 6.7% of the heaviest white male drinkers consume 33% of the overall consumption; in the upshifted distribution for the same group, 10.2% consume this proportion [36]. It is thus possible that a large part of the undercoverage is due to sampling schemes, and hence there might be less systematic underreporting among respondents than it appears. However, as laid out above, it is unlikely that sampling explains all of the undercoverage. There is some evidence for individual underreporting as well [11].

The third explanation is that the assumption that the sales/production figures are the gold standard might be wrong. This explanation seems implausible except in circumstances where there is large unrecorded consumption. Where there is much unrecorded consumption, including that in the sales/production estimate would of course increase the differences with estimates from surveys.

It is unlikely that there is much alcohol measured in the sales/production figures that is not in fact consumed. Why would consumers pay for goods that would be wasted, e.g., as in the case of Canada cited above, a wastage of 60% to 70%? Such behavior would be contradictory to evidence from economics. Clearly, some alcohol bought is not consumed due to spillage. However, according to industry experts, this spillage should amount to less than 10%. Other alcohol may also be stocked rather than consumed in the year of purchase, but overall across regions and years, this should cancel out. Thus, the assumption of per capita consumption derived from aggregate statistics such as sales and/or production being the best estimate for overall volume of consumption seems justified. Exploring the factors involved in undercoverage by surveys should be a research priority in coming years. It seems wasteful to conduct hundreds of annual surveys with questions about alcohol in high-income countries without knowledge of why these surveys typically cover only proportions between 30% to 60% of per capita consumption. The assumption made in the present analysis of constant undercoverage in different population segments also needs to be tested and replaced by empirical estimates of differential undercoverage.

However, irrespective of reasons for undercoverage, triangulation is necessary for the comparison of alcohol exposure. If surveys are compared to each other, they should have their proportions of coverage standardized in the same way that disease rates are standardized to correct for population distributions. It would also appear irrational to continue the comparisons of surveys in which the underlying coverage rates starkly differ as the results of such comparisons are not interpretable. This reasoning is independent of the level of upshifting chosen. Based on the uncertainty about the degree of underreporting in medical epidemiological studies, we suggest the routine application of sensitivity analyses using 100%, 90%, and 80% of per capita consumption as the target levels when the actual level of population consumption is important. This method will be used not only in the CRA 2005, but, together with new empirically determined disability weights, also in the ongoing US Burden of Disease study [37].

A final point concerns the assumptions made in the upshifting. First, we assumed the proportion of abstainers and ex-drinkers to be exactly as assessed by the survey. Unfortunately, there is no better information available on which different estimates could be based. The medical epidemiological literature is of no help here, as cohorts get selected based on their potential to be followed up, and this may yield some proportions of abstainers that are not at all representative of the general population. Second, we assumed that proportions by sex and age are correctly estimated by the survey. Again, there are no better data currently available. We can only speculate if and how the inclusion of nonhousehold members shifts the proportions of alcohol consumed. More research is necessary on such populations to estimate the bias introduced by relying only on households in the sampling frame.

## Conclusion

Overall, the chosen methods for estimating alcohol exposure for population health proved feasible and seemed justified based on current knowledge. Further work is needed for refinement of methods and validation of assumptions.

## Conflict of interest

The authors declare that they have no competing interests.

## Authors' contributions

JR supervised all aspects of the work and wrote the first draft. TK programmed most of the statistical tests and contributed to the writing. GG Jr contributed to the programming, did part of the statistical analyses, and contributed to the writing. FS and BG did part of the statistical analyses and contributed to the writing. GG Sr. contributed to the programming, helped design the study, and contributed to the writing. All authors read and approved the final manuscript.

## Supplementary Material

Additional file 1 Description of key parameters on volume of alcohol exposure from NESARC (weighted to be representative for the population). Click here for file
