# Supplementary material for: Statistical modeling of volume of alcohol exposure for epidemiological studies of population health: the US example
Source: Popul Health Metr. 2010 Mar 4;8:3. doi: 10.1186/1478-7954-8-3 (PMC2841092; doi:10.1186/1478-7954-8-3)
Supplement: Additional file 1 — Description of key parameters on volume of alcohol exposure from NESARC (weighted to be representative for the population). [file 1478-7954-8-3-S1.PDF]

Additional file 1: Description of key parameters on volume of alcohol exposure from NESARC (weighted to be representative for the population)

Sex	Ethnicity	Age category	N total	N lifetime abstainer	% abstainers, weighted	N exdrinker	% ex drinker, weighted	N drinker	Average alcohol consumption in g/day	SE of average consumption	Average consumption drinkers in g/day	SE of average consumption
Men	White	18-34	2784	282	10.1	225	8.1	2277	21.862	1.167	26.795	1.446
Men	White	35-54	4413	315	7.3	661	15.4	3437	15.920	0.784	20.654	1.014
Men	White	55+	3648	396	10.9	969	26.0	2283	9.868	0.493	15.739	0.750
**Men**	**White**	**all ages**	**10845**	**993**	**9.2**	**1855**	**16.5**	**7997**	**15.792**	**0.511**	**21.330**	**0.702**
Men	Black	18-34	906	174	19.5	86	9.4	646	18.037	2.307	25.402	3.067
Men	Black	35-54	1277	157	12.9	288	23.1	832	17.795	1.994	28.000	2.935
Men	Black	55+	858	152	17.6	329	37.8	377	10.463	1.524	23.646	3.369
**Men**	**Black**	**all ages**	**3041**	**483**	**16.3**	**703**	**21.0**	**1855**	**16.365**	**1.311**	**26.257**	**1.915**
Men	Native	18-34	84	11	11.5	8	11.4	65	28.722	9.465	37.443	12.129
Men	Native	35-54	140	16	11.8	29	22.8	95	20.042	5.282	30.781	7.916
Men	Native	55+	90	8	8.8	34	38.4	48	7.299	2.206	14.520	4.313
**Men**	**Native**	**all ages**	**314**	**35**	**11.0**	**71**	**23.5**	**208**	**19.391**	**3.866**	**29.942**	**5.795**
Men	Asian/Pac. Islander	18-34	262	61	25.3	21	6.8	180	9.337	1.893	13.783	2.593
Men	Asian/Pac. Islander	35-54	233	58	24.2	33	17.0	142	7.089	1.531	12.069	2.725
Men	Asian/Pac. Islander	55+	101	39	37.7	14	9.8	48	3.254	1.551	6.251	2.788
**Men**	**Asian/Pac. Islander**	**all ages**	**596**	**158**	**27.3**	**68**	**11.2**	**370**	**7.328**	**1.073**	**11.942**	**1.716**
Men	Hispanic	18-34	1615	272	16.4	138	9.9	1205	14.038	1.359	19.148	1.838
Men	Hispanic	35-54	1439	185	14.4	193	15.1	1061	19.436	6.027	27.658	8.430
Men	Hispanic	55+	668	118	17.4	179	27.8	371	6.784	0.760	12.457	1.281
**Men**	**Hispanic**	**all ages**	**3722**	**575**	**15.8**	**510**	**14.2**	**2637**	**15.028**	**2.214**	**21.572**	**3.154**
**Men**	**total**	**all ages**	**18518**	**2244**		**3207**		**13067**	**15.457**	**0.521**	**21.600**	**0.729**

Sex	Ethnicity	Age category	N total	N lifetime abstainer	% abstainers, weighted	N exdrinker	% ex drinker, weighted	N drinker	Average alcohol consumption in g/day	SE of average consumption	Average consumption drinkers in g/day	SE of average consumption
Women	White	18-34	3433	428	13.3	319	9.1	2686	7.120	0.467	9.192	0.604
Women	White	35-54	4944	540	11.8	833	17.3	3571	5.336	0.246	7.534	0.339
Women	White	55+	5285	1453	26.7	1354	24.7	2478	3.197	0.178	6.619	0.335
**Women**	**White**	**all ages**	**13662**	**2421**	**17.3**	**2506**	**17.6**	**8735**	**5.085**	**0.178**	**7.832**	**0.271**
Women	Black	18-34	1567	434	30.3	220	13.6	913	5.856	0.828	10.511	1.477
Women	Black	35-54	2061	507	26.7	508	24.3	1046	5.848	1.445	11.993	2.973
Women	Black	55+	1576	626	40.0	579	34.6	371	1.636	0.445	6.522	1.691
**Women**	**Black**	**all ages**	**5204**	**1567**	**31.2**	**1307**	**22.9**	**2330**	**4.850**	**0.662**	**10.631**	**1.483**
Women	Native	18-34	112	25	23.0	16	15.3	71	15.959	7.111	26.027	11.518
Women	Native	35-54	157	21	16.6	43	29.1	93	7.827	3.148	14.486	5.434
Women	Native	55+	118	33	32.0	41	30.7	44	3.792	1.870	10.523	4.710
**Women**	**Native**	**all ages**	**387**	**79**	**22.8**	**100**	**25.6**	**208**	**9.045**	**2.528**	**17.695**	**4.740**
Women	Asian/Pac. Islander	18-34	286	107	41.1	34	11.9	145	6.082	2.162	12.939	4.654
Women	Asian/Pac. Islander	35-54	303	149	53.3	34	12.1	120	1.003	0.189	2.892	0.503
Women	Asian/Pac. Islander	55+	147	87	60.3	31	20.4	29	0.246	0.106	1.287	0.524
**Women**	**Asian/Pac. Islander**	**all ages**	**736**	**343**	**50.1**	**99**	**13.7**	**294**	**2.763**	**0.831**	**7.662**	**2.272**
Women	Hispanic	18-34	1909	620	34.2	226	12.2	1063	4.371	0.937	8.169	1.771
Women	Hispanic	35-54	1748	545	33.0	263	14.9	940	6.789	3.567	13.073	6.844
Women	Hispanic	55+	929	447	46.9	173	19.7	309	1.881	0.635	5.658	1.905
**Women**	**Hispanic**	**all ages**	**4586**	**1612**	**36.0**	**662**	**14.5**	**2312**	**4.837**	**1.407**	**9.790**	**2.852**
**Women**	**Total**	**all ages**	**24575**	**6022**		**4674**		**13879**	**5.012**	**0.231**	**8.442**	**0.386**

* The categories white and black exclude Hispanics.
